# Neuromuscular Characteristics of Female Futsal Players: A Systematic Review

**DOI:** 10.3390/sports14030098

**Published:** 2026-03-03

**Authors:** Catarina Marques, Miguel Rebelo, João Serrano, Hélder Fonseca

**Affiliations:** 1 Research Centre in Physical Activity, Health and Leisure (CIAFEL), Faculty of Sport, University of Porto (FADE-UP), 4200-450 Porto, Portugal; hfonseca@fade.up.pt; 2 Department of Sports and Well-Being, Polytechnic Institute of Castelo Branco, 6000-266 Castelo Branco, Portugal; miguel.rebelo@ipcb.pt (M.R.); j.serrano@ipcb.pt (J.S.); 3 SPRINT Sport Physical Activity and Health Research & Innovation Center, 6000-084 Castelo Branco, Portugal

**Keywords:** indoor soccer, strength and conditioning, physical capacities, sports performance, team sports

## Abstract

Background: This systematic review aimed to analyze and synthesize the available evidence on the neuromuscular profile of female futsal players. Methods: The review was conducted in accordance with PRISMA guidelines and registered with PROSPERO (CRD420251055503). PubMed, Scopus and SPORTDiscus databases were searched until May 2025. The eligibility criteria were defined using the PECOS strategy. The methodological quality of the included studies was evaluated using the Downs and Black modified version. Results: Twenty-three studies were included, covering a total of 433 female players between the ages of 12 and 27. Muscle strength was assessed mainly using an isokinetic dynamometer, jumping ability using the countermovement jump and squat jump, sprinting using the 10 m, 20 m and 30 m tests and agility and change of direction (CoD) using the Illinois agility test. Elite players generally showed a better performance in agility and CoD tests compared to lower-level players. Conclusions: This review provides a comprehensive overview of the neuromuscular profile of female futsal players and highlights trends related to the competitive level. These findings may support evidence-based practices for performance evaluation, training and injury prevention. More studies are needed to standardize methods and understand differences between competitive levels.

## 1. Introduction

Futsal, considered the indoor version of five-a-side soccer, originated in 1930 and is officially regulated by the Fédération Internationale de Football Association (FIFA) [1]. It is characterized as an intermittent, high-intensity and unpredictable sport that requires explosive technical and tactical actions under temporal and spatial pressure, such as sprints, jumps, successive accelerations and rapid changes in direction [2]. It is played on a court measuring approximately 40 m × 20 m with 3 m × 2 m goals, between two teams of five players (four field players and a goalkeeper) during two 20 min halves, where the timer is stopped whenever the ball leaves the field of play or there is a foul or a stoppage in play [3,4].

Compared to soccer, technical proficiency in futsal is largely influenced by the specifics of the sport, such as the small size of the ball with 30% less bounce, which forces players to develop more precise control and quick movement of the ball on the ground [5]. In addition, the small size of the court and the dynamic of unlimited substitutions throughout the game require players to make quick decisions, maintain a high speed of execution and be able to repeat sprints under high pressure during offensive and defensive phases [5]. In women’s futsal, match-analysis evidence indicates high internal and external loads, with performance often declining in second halves, and agility and sprint-related capacities emerge as relevant performance components across competitive levels, though measurement heterogeneity remains a challenge [6].

Simultaneously, performance in futsal depends on neuromuscular qualities, particularly high anaerobic power and the ability to generate rapid force [7,8]. In female players, recent evidence suggests that acceleration, repeated short explosive actions and the ability to rapidly have a change of direction (CoD) are especially relevant, aligning with the specificities of the sport (a small field, frequent accelerations/decelerations, recurring 1 vs. 1 duels) [6]. These demands make strength, power and the CoD capacity important for high-level performance [9]. Nevertheless, the specific literature on women advises caution against overemphasizing the maximum speed alone, as differences in the competitive level in women often manifest themselves through acceleration/deceleration and movement efficiency rather than the maximum speed itself [10]. A CoD in futsal is commonly assessed with field tests such as the Illinois Agility Test (IAT), 505/Modified-505, *t*-test and other short-distance protocols which incorporate rapid decelerations and re-accelerations [6].

Furthermore, sex-specific considerations are essential to contextualize the neuromuscular performance. Women differ from men in several aspects such as their absolute strength and power, neuromuscular control, fatigue characteristics and injury risk profiles [11,12]. For example, research shows that women may exhibit similar or specific patterns of neuromuscular fatigue compared to men, with differences depending on the type of contraction, load and muscle groups tested [13]. Hormonal fluctuations throughout the menstrual cycle may influence strength, neuromuscular function and perceived exertion, although evidence remains heterogeneous and highly individualized [14,15]. The epidemiology of injuries also differs substantially, as female athletes have a higher prevalence of ligament injuries such as the anterior cruciate ligament [14,16], with non-contact mechanisms often associated with pivoting, landing and rapid CoD, movement patterns that are highly relevant to futsal [17]. These sex-specific factors reinforce that female athletes cannot be considered as reduced versions of male players and should be studied in their own physiological and biomechanical contexts.

Despite the significant growth in women’s futsal worldwide [18,19], much of the research in the broader futsal literature remains focused on male players and, therefore, evidence remains limited in several neuromuscular domains [7,19]. In particular, isokinetic strength profiles, lower limb power, acceleration in the initial phase of sprinting and performance in CoD have been examined in relatively few studies, often with small samples and heterogeneous testing protocols [6]. This scarcity limits the development of robust normative references, evidence-based training prescriptions, and injury prevention strategies in women’s futsal, reducing the applied impact of the available research [20].

Therefore, detailed and systematic knowledge of neuromuscular characteristics can be of great interest to coaches, physical trainers, and physiotherapists, as it allows them to identify talent [21], reduce the risk of injury [17], optimize performance [7], periodize training loads, and individualize interventions according to one’s position on the field or competitive level [22,23,24]. With this in mind, the present review sought to (1) summarize the available evidence describing the neuromuscular characteristics of female futsal players, (2) identify common performance trends and reported normative ranges and (3) examine potential differences between competitive levels. Importantly, we note that any conclusions regarding differences in competitive levels should be interpreted with caution, given the marked heterogeneity in study designs, measurement protocols and sample characteristics.

## 2. Materials and Methods

### 2.1. Study Design

This systematic review on the neuromuscular profile of female futsal players was conducted in accordance with the PRISMA (Preferred Reporting Items for Systematic Reviews and Meta-Analyses) guidelines [25] and was registered in the International Prospective Register of Systematic Reviews (PROSPERO), under the number CRD420251055503, in order to guarantee the transparency and methodological systematization of the process. All included studies explicitly reported ethical approval in accordance with the Declaration of Helsinki.

### 2.2. Search Strategy

The databases used for this systematic review were PubMed, Scopus and SPORTDiscus, as they offer the most comprehensive coverage of research in sports science, human performance and applied physiology, ensuring that potentially all relevant studies on futsal players could be retrieved. The searches were carried out between April and May 2025, with no restrictions regarding the year of publication. The search strategy combined keywords or equivalent terms from the Medical Subject Heading (MeSH) related to women’s futsal and neuromuscular variables, using Boolean operators to maximize the search effectiveness. The complete database-specific search strings are provided in Supplementary File S1.

### 2.3. Eligibility Criteria

The eligibility criteria were defined based on the PECOS strategy [26], as described in Table 1. The inclusion of adolescent and adult players is justified by the limited availability of studies on women’s futsal, which often combine age groups due to small sample sizes. Original peer-reviewed studies were included, published in English, Portuguese or Spanish, involving female futsal players (adolescents or adults) and presenting variables related to neuromuscular characteristics. Studies were excluded if: (i) the participants’ sex was not identified; (ii) they included men or results grouped with male athletes; (iii) they presented results mixed with other sports or without specific data on female futsal players; or (iv) they were literature reviews, commentaries, editorials, conference papers or abstracts from scientific events.

### 2.4. Study Selection

Two authors (CM and MR) carried out the literature search and independently analyzed the titles and abstracts based on the previously defined eligibility criteria. All identified studies were initially exported to EndNote 20.3 (Clarivate Analytics, Philadelphia, PA, USA) to remove duplicates. Subsequently, they were imported into Rayyan (https://www.rayyan.ai/, accessed on 15 May 2025), a free online systematic review management platform [27]. Rayyan was used to screen the studies by title and abstract, allowing the PECOS strategy to be applied. This tool has already been used in other systematic reviews and is widely recommended for its efficiency and reliability in the screening process by multiple reviewers [28,29]. In addition to database screening, the reference lists of all included articles were manually searched to identify any potential additional relevant studies. All records were screened independently by two reviewers. Any inconsistencies in the study selection or data extraction were discussed and resolved by consensus. Whenever necessary, a third reviewer was involved to arbitrate disagreements.

### 2.5. Data Extraction

Two reviewers (CM and MR) independently extracted the data from the included studies using a standardized pre-prepared table in Microsoft Excel. The following information was collected: the study information (authors and the year of publication), sample characteristics (the number of participants, age, height, body mass and competitive level), the neuromuscular variables analyzed (muscle strength, jumping performance, speed, agility and CoD), evaluation protocols and main results (mean, standard deviation and measurement units).

Competitive levels were standardized as follows: elite (top national league), sub-elite (second or third national divisions), university, regional/amateur and unspecified. These definitions were applied consistently across all included studies to improve the comparability.

As one of the aims of this review was to describe the neuromuscular profile of female futsal players, in the studies in which interventions were applied (e.g., training programs or interventions), only the baseline values were recorded in order to ensure comparability between studies.

### 2.6. Methodological Quality Assessment

The methodological quality of the included studies was independently assessed by two reviewers (CM and MR). Following previous systematic reviews that applied adapted versions of the Downs and Black checklist to non-interventional study designs [29,30,31,32,33], we selected 12 items from the original scale because they correspond to methodological domains appropriate for the predominantly cross-sectional and observational studies included in this review. Specifically, the selected items evaluate the clarity of reporting (items 1, 2, 3 and 4), external validity (items 6 and 7), internal validity related to measurement bias (items 10, 11 and 12) and confounding or selection bias (items 16, 18 and 20). Items pertaining to randomization, intervention integrity or blinding were excluded as they are not applicable to the study designs analyzed.

Before independently scoring the studies, the reviewers conducted a short calibration process to ensure the consistent interpretation of the criteria. Each item was rated as “0” when information was absent or unclear and “1” when explicitly reported. The maximum achievable score was 12, with studies categorized as a high (10–12), moderate (7–9), low (4–6) or very low (0–3) methodological quality. Discrepancies between reviewers were resolved through discussion and a third reviewer was available for arbitration when necessary. No study was excluded based on the methodological quality.

### 2.7. Statistical Analysis

A meta-analysis was not performed because of the heterogeneity of the study designs, measurement protocols and variables which prevented statistical pooling. However, since only baseline data were extracted, comparability across studies was improved, allowing for a structured descriptive and narrative synthesis of the findings. All results are presented as the mean ± standard deviation (SD) and grouped by variable and competitive level when possible.

## 3. Results

### 3.1. Study Selection and Study Characteristics

**Figure 1:**
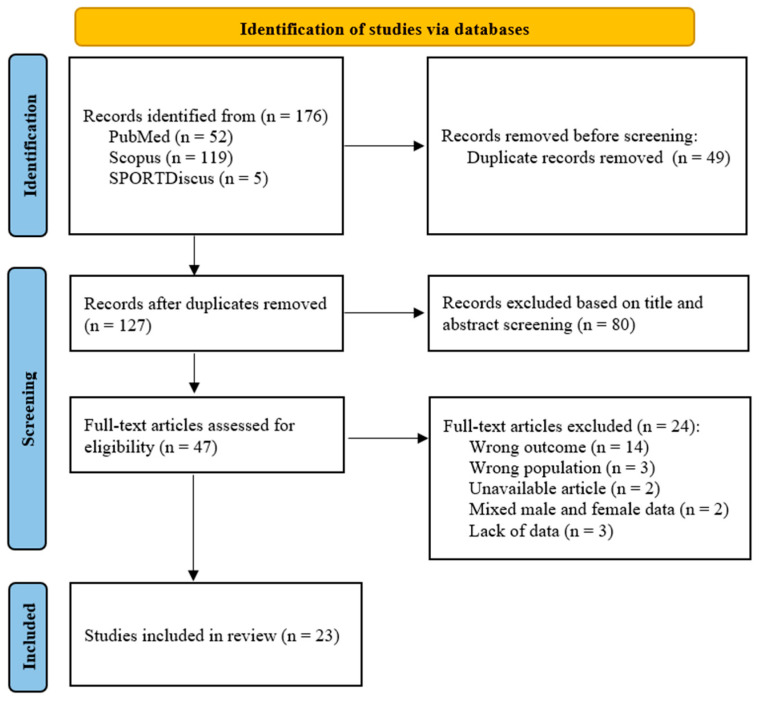
PRISMA flow diagram.

Figure 1 shows the PRISMA flowchart of the process of identification, screening, eligibility and inclusion of studies. The completed PRISMA check-list is provided in the Supplementary Files S2 and S3. The literature search in the databases initially resulted in 176 studies. After removing 49 duplicates, 127 studies were analyzed by title and abstract, of which 47 articles were considered potentially eligible. After reading the full text, 24 articles were excluded because they did not meet the inclusion criteria. Thus, 23 studies published between 2010 and 2025 were included in this systematic review, involving a total sample of 433 female futsal players.

The detailed characterization of the samples is shown in Table 2. In all the studies, the age of the players was between 12 and 26, their height was between 154 and 167 cm and their body mass was between 49 and 65 kg. With regard to their competitive level, 11 studies analyzed elite players, 4 studies sub-elite players, 3 studies university players, 1 study regional (amateur) players and 5 studies made no reference to the competitive level of the players (unspecified).

### 3.2. Methodological Quality

The scores and categorization of the studies based on the assessment of the methodological quality are shown in Table 3, ranging from 4 to 12 (low to high, respectively) of the 12 items analyzed.

### 3.3. Neuromuscular Characteristics

Regarding the neuromuscular variables analyzed, 5 studies evaluated muscle strength, 15 studies lower limbs power (jump ability), 16 studies sprint performance and 13 studies investigated agility and CoD. At the same time, some studies assessed more than one neuromuscular variable and were, therefore, considered in multiple subcategories of analysis. Across all neuromuscular variables, cross-study comparisons are limited by substantial methodological heterogeneity, including differences in the testing protocols, measurement units, equipment, familiarization procedures and timing systems. Due to this variability, all interpretations of performance differences should be considered with caution and any observed patterns must be viewed as descriptive rather than definitive.

#### 3.3.1. Strength Capability

Muscle strength was analyzed in five studies [7,37,44,51,53], mainly through the isokinetic muscle testing of the knee extensors and flexors (Table 4). In Teixeira et al. [37], isokinetic strength was assessed in the knee extensors of the dominant leg (“kicking leg”) during concentric and eccentric actions at 60°/s. Across the studies, the absolute peak torque values for knee extensors and flexors generally fell within similar ranges and did not consistently differ between competitive levels, a pattern also observed by Ramos-Campo et al. [7]. The interpretation of the differences across the studies is further limited by the substantial methodological heterogeneity in the testing protocols, including variations in the angular velocity (60°/s vs. 180°/s), testing procedures, warm-up and familiarization routines and differences in the measurement indicators (absolute torque vs. relative torque). The ratios between the hamstring and quadriceps (H/Q) have been reported in some studies and typically range between ~0.5 and 0.6 [7], suggesting an optimal muscle balance [24].

Regarding functional strength assessments, only one study reported 1RM squat values [44] and another assessed isometric back and leg strength using a portable dynamometer [53]. Due to the limited number of studies that have utilized multi-joint or task-specific strength tests that reflect the integrated movement patterns of futsal, the applicability of current findings to the actual demands of matches remains limited.

It is important to note that Ahmadi et al. [51] reported strength results as relative values of maximum torque (%), normalized for body mass, rather than absolute torque (Nm). Similarly, Atakan et al. [53] reported isometric back and leg strength in kilograms, reflecting the output of the specific dynamometer used. Purnamasari et al. [44] also reported the results in kilograms. As these metrics differ conceptually from absolute isokinetic torque, they are not directly comparable to the values in Nm reported in other studies and should, therefore, be interpreted independently.

#### 3.3.2. Jumping Ability

The jump performance was assessed in fifteen studies using a variety of protocols, with CMJ being the most frequently used measure (Table 5). Overall, CMJ values showed moderate variability across studies, but it was still possible to identify general performance patterns at all competitive levels. Elite players typically had higher CMJ heights, with most values clustered between 26.7 and 35.6 cm, while sub-elite (23.9 to 25.7 cm) and university (25.6 to 26.7 cm) players tended to score slightly lower. However, this trend was not entirely consistent, as some studies reported overlapping ranges between levels. For example, although Souglis et al. [40] reported one of the highest CMJ values (35.6 ± 2.9 cm), Ramos-Campo et al. [7] found no statistically significant differences between elite and sub-elite players.

These inconsistencies are likely influenced by heterogeneity in testing procedures, including variations in the jump technique (with or without arm swing), the equipment used for the measurement, warm-up protocols, the number of attempts and familiarity with the test. Such methodological variability limits the comparability of absolute values and may partly explain the lack of clear differences between competitive levels.

Other protocols were used, such as the squat jump (SJ), drop vertical jump (DVJ), horizontal jump (HJ), vertical jump (VJ), Abalakov jump (ABK) and broad jump (BJ), but these were included less frequently. As a result, while they provide additional insight into lower-limb power characteristics, their limited use prevents a meaningful cross-study comparison or synthesis. When examined collectively, these tests reinforce the notion that the jump performance contributes to neuromuscular profiling, yet the current evidence does not consistently demonstrate differences between competitive levels, largely due to methodological variability and heterogeneous testing procedures.

#### 3.3.3. Sprint Performance

The sprint performance was assessed in sixteen studies using a range of linear sprint distances and field-based speed protocols [5,7,34,35,36,37,38,39,40,43,44,47,48,49,53,54] (Table 6). Although sprint times varied considerably across studies, some general performance patterns can be identified. In most investigations, elite players tended to achieve slightly faster sprint times than their sub-elite or university counterparts, particularly over short distances (5–20 m), which reflects the high frequency of accelerations characteristic of futsal match play. However, these trends were not consistent across all studies, and Ramos-Campo et al. [7] reported no significant differences between elite and sub-elite groups.

A major factor-limiting cross-study comparability was the substantial methodological heterogeneity in the sprint testing procedures. Studies differed in their sprint distance (5 to 40 m), starting procedures (static vs. flying start), timing systems (manual timing, photocells or radar), surface type and warm-up standardization. In addition, some studies reported sprint outcomes as velocity (m/s) or endurance-related speed metrics (km/h) rather than time (s). This variation in units further complicates a direct comparison between studies and likely contributes to the lack of consistent differences at the competitive level.

#### 3.3.4. Change of Direction Ability and Agility

CoD ability and agility were examined in thirteen studies [5,7,36,39,41,42,43,45,47,49,52,53,54] using a variety of field-based tests, including the Illinois Agility Test (IAT), 505 and Modified 505 tests, *t*-test variations, L-Run, V-Cut and reactive agility protocols (Table 7). Despite the diversity of the methods, the CoD performance was the neuromuscular dimension that most consistently differentiated players across competitive levels.

Several studies reported a slightly superior CoD performance in elite players compared with sub-elite or amateur counterparts. For example, Ramos-Campo et al. [7] observed faster agility test completion times in elite players relative to sub-elite players (elite: 5.5 s; sub-elite: 5.7 s), suggesting that agility-related capacities may partially distinguish higher-level athletes. However, these differences were not universal and some studies reported overlapping performance ranges across competitive groups.

Similar to other neuromuscular variables, the methodological heterogeneity limits the interpretation of the CoD and agility results. For example, differences in the inclusion of planned CoD tests (e.g., IAT, 505) and reactive agility assessments, device accuracy (e.g., photocells versus manual timing) and test surfaces. This variability reduces the direct comparability between studies and may obscure the magnitude of true performance differences.

Overall, CoD and agility show the most consistent evidence of potential differences between competitive levels in women’s futsal; however, these patterns should be interpreted with caution, as the evidence does not consistently demonstrate clear discrimination due to heterogeneous protocols and limited sample sizes.

## 4. Discussion

The main objective of this systematic review was to characterize the neuromuscular profile of female futsal players and to identify potential differences between competitive levels. The synthesis of the available evidence allowed us to describe performance patterns across several neuromuscular domains while highlighting important methodological limitations and gaps in the literature.

While some performance comparisons with female football players provide useful reference points, particularly given the limited literature available on women’s futsal, direct equivalence should not be assumed. Futsal imposes distinct neuromuscular demands due to its smaller playing space, higher acceleration density and substantially greater frequency of rapid CoD actions. These characteristics create a performance profile that differs meaningfully from football, even though certain benchmarks from female football may still help contextualize findings in areas where futsal-specific evidence remains scarce.

According to the studies included, muscle strength in futsal players has been predominantly assessed using isokinetic dynamometry, with maximum concentric torque values of the knee extensors ranging from 131 to 149 Nm in elite players [7,37]. When compared to athletes from other intermittent team sports, such as women’s football, similar magnitudes are reported [55,56,57], suggesting that the neuromuscular strength profile of futsal players falls within the expected range for multidirectional sports. However, the only available multi-joint strength measure (squat 1RM) indicated slightly lower relative strength values compared with female football players [58]. Furthermore, current evidence does not consistently demonstrate strength differences between competitive levels [7], likely due to small samples, heterogeneous protocols and the limited ecological relevance of isolated isokinetic measures. Future research should implement standardized and functional strength assessments to establish normative ranges in women’s futsal.

The jump performance, assessed mainly through the CMJ, showed values between ~26 and 36 cm [7,37,40,41,42,43,48,49,50,52], slightly below those recorded for elite football players [59], but similar to ranges found in semi-professional football players [58,60]. Nevertheless, the methodological inconsistency across jump protocols, equipment and test conditions restricts the comparability across studies and may explain the lack of consistent differences between competitive levels, a pattern also observed in women’s football [61].

The sprint capacity in female futsal players (1.60–2.06 s in the 10 m; 2.98–5.33 s in the 20 m; 4.16–5.77 s in the 30 m) [7,36,47,53] is generally similar to values reported in female football players [57,58,62,63]. Although this similarity is expected, given the intermittent and explosive nature of both sports, no consistent differences were observed between competitive levels in futsal, contrasting with other studies [63]. This may relate to the highly technical and tactical nature of futsal, where the acceleration ability, decision making under pressure and ball control may be more decisive than the maximal sprint speed. Importantly, the substantial methodological heterogeneity (sprint distances, timing systems and unit formats) further limits cross-study comparisons.

CoD and agility were the neuromuscular variables that most consistently suggested potential differences between competitive levels. Elite players tended to complete CoD tasks faster than sub-elite players [7] and also performed better than football players in similar tests [64]. However, it is important to note that assessing agility in futsal requires a clear distinction between planned CoD, which reflects mechanical efficiency and stopping-propulsion ability, measured by tests such as the IAT or the 505, and reactive agility, which involves responding to unpredictable external stimuli and requires perceptual–cognitive processing [5,9]. Evidence from female players further reinforces the distinction between these two concepts [6,18,19]. Benvenuti et al. [5] reported that futsal players were significantly faster than football players in reactive agility and exhibited shorter decision times, probably reflecting the greater spatial constraints of the sport, the faster pace of the game and the greater information load. Interestingly, no differences were found between the sports in planned agility tests, suggesting that only reactive agility tasks capture the specific perceptual–motor demands of futsal, while planned CoD mainly assesses the physical qualities of the players. Most studies included in this review assessed only planned CoD, which limits the interpretation of agility in a manner representative of real match demands. Future research should incorporate ecologically valid, stimulus-based protocols to better characterize this capacity and its relevance to competitive success [9].

In summary, the neuromuscular profile of female futsal players seems to be broadly aligned with that of athletes from other intermittent, high-intensity team sports. However, evidence suggests that the CoD capacity, and particularly its reactive component, may represent a more specific and potentially discriminatory characteristic in women’s futsal. This has direct implications for talent identification, individualized training prescription, injury prevention strategies and performance monitoring. However, the small number of studies available, combined with significant methodological heterogeneity, prevents definitive conclusions. Future research should employ standardized and ecologically valid test batteries, differentiate between planned and reactive agility tasks and explore the specific neuromuscular demands of each position.

## 5. Limitations

Despite the methodological rigor adopted throughout this systematic review, some aspects should be recognized as limitations and taken into account when interpreting the results.

Primarily, the general lack of studies on the neuromuscular profile of female futsal players limited the general and more detailed analysis, especially regarding comparisons between competitive levels. Most of the studies included evaluated only small samples and often made no distinction between competitive levels. This, however, highlights the need of more research in this area. Another limitation relates to the lack of studies analyzing neuromuscular characteristics according to playing positions. Although positional demands differ in futsal, only a very small number of studies have examined performance variations between goalkeepers, defenders, wingers or pivots. This gap limits the ability to establish positional neuromuscular profiles in women’s futsal.

In addition, the heterogeneity between the protocols used in the physical tests made it difficult to make direct comparisons between studies. Highly different methodologies were identified for assessing the muscle strength, jumping ability, sprinting, agility and CoD, which made it impossible to carry out a meta-analysis. In addition, not all studies provided exact values (e.g., standard deviations and consistent units), sometimes compromising the interpretation of the results.

## 6. Conclusions

This systematic review synthesized the available evidence on the neuromuscular profile of female futsal players. Overall, the findings suggest that their neuromuscular characteristics are broadly comparable to those reported in other intermittent, high-intensity team sports. Among the variables analyzed, the CoD ability showed the most consistent indications of potential differences between competitive levels; however, these trends should be interpreted cautiously due to the limited number of studies, small sample sizes and the substantial methodological heterogeneity observed. In contrast, the muscle strength, jumping performance and sprint capacity did not demonstrate consistent or robust differences across competitive levels.

From a practical point of view, this review provides a structured overview of the current evidence on the neuromuscular profile of female futsal players and its findings may support coaches, performance staff and health professionals in planning neuromuscular training programs that emphasize strength, power, sprint ability and agility, skills that seem essential in futsal. The synthesized data may also assist in talent identification, load management and the development of targeted performance and injury prevention strategies aligned with the sport’s specific demands.

Finally, this review reinforces the relevance of the continued investigation of the neuromuscular capacities of female futsal players, particularly through studies with more rigorous methodological designs, larger and more representative samples and ecologically valid testing procedures. This will provide a solid and specific evidence base that can contribute both to the sustainable growth of this sport at all levels and to greater equity in the production and application of practical knowledge between sexes.

## Figures and Tables

**Table 1:** PECOS strategy.

PECOS	Description
Participants	Female athletes (adolescents and adults) who play futsal at any competitive level (elite, sub-elite, amateur, university or other).
Exposure	Regular participation in futsal training and competition.
Comparisons	Between competitive levels (when available).
Outcomes	Objective physical performance tests as muscle strength, jumping, sprinting, agility and CoD.
Study design	Experimental, quasi-experimental, cohort, cross sectional, descriptive and observational studies.

**Table 2:** Characterization of the included studies.

Study	Participants	Competitive Level
Benvenuti et al. [5]	18 (22.9 ± 2.8 years, 163 ± 3.6 cm, 56.2 ± 5.9 kg)	Regional
Ramos-Campo et al. [7]	27 (elite: *n* = 14, 21.1 ± 2.3 years, 163.5 ± 4.2 cm, 61.8 ± 4.6 kg; sub-elite: *n* = 13, 21.8 ± 2.5 years, 166.2 ± 3.1 cm, 65.1 ± 1.6 kg)	Elite and sub-elite
Barbero-Alvarez et al. [34]	14 (21.2 ± 4.0 years, 161 ± 5.0 cm, 58.6 ± 5.6 kg)	Elite
Valladares-Rodríguez et al. [35]	14 (23.3 ± 4.5 years, 165.8 ± 6.2 cm, 61.7 ± 5.5 kg)	Elite
Ünveren [36]	35 (20.85 ± 1.88 years, 166.85 ± 4.57 cm, 61.74 ± 9.07 kg)	University
Teixeira et al. [37]	14 (18.71 ± 1.94 years, 160.57 ± 3.86 cm, 59.23 ± 8.00 kg)	Elite
Teixeira et al. [38]	16 (19.2 ± 2.0 years, 161.5 ± 4.6 cm, 58.7 ± 8.0 kg)	Elite
Supriadi et al. [39]	10 (NR)	Unspecified
Souglis et al. [40]	34 (22.6 ± 2.1 years, 167.4 ± 3.6 cm, 57.5 ± 3.0 kg)	Elite
Söğüt et al. [41]	33 (12.2 ± 0.9 years, 156.5 ± 9.2 cm, 49.0 ± 10.1 kg)	Unspecified
Santos et al. [42]	13 (24.1 ± 3.7 years, 1.61 ± 0.4 m, 63.6 ± 8.0 kg)	Unspecified
Ruiz-Pérez et al. [43]	22 (21.45 ± 4.97 years, 1.63 ± 0.07 m, 58.75 ± 6.93 kg)	Elite
Purnamasari et al. [44]	10 (19.1 ± 1.101 years, 1.54 ± 0.037 m, 50.37 ± 6.554 kg)	Unspecified
Patti et al. [45]	15 (26.80 ± 6.52 years, 165.07 ± 7.61 cm, 63.31 ± 5.30 kg)	Elite
Neves da Silva et al. [46]	10 (19.5 ± 1.4 years, 165.1 ± 5.8 cm, 62.5 ± 8.8 kg)	University
Doewes et al. [47]	20 (18.30 ± 1.06 years, 160.80 ± 3.68 cm, 50.90 ± 1.73 kg)	Unspecified
Lucas et al. [48]	25 (22.03 ± 2.80 years, 161.19 ± 0.05 cm, 57.98 ± 8.94 kg)	University
Castillo et al. [49]	12 (25.17 ± 4.75 years, 1.64 ± 0.05 m, 59.79 ± 6.36 kg)	Elite
Canduela Valle et al. [50]	8 (25.78 ± 6.44 years, 165.0 ± 7.07 cm, 60.54 ± 7.31 kg)	Sub-elite
Ahmadi et al. [51]	15 (22.93 ± 0.54 years, 159.60 ± 1.16 cm, 56.95 ± 1.79 kg)	Elite
Albalad-Aiguabella et al. [52]	41 (22.69 ± 4.76 years, 161.28 ± 6.03 cm, 62.20 ± 10.34 kg)	Sub-elite
Atakan et al. [53]	15 (20.7 ± 1.33 years, 165.94 ± 5.82 cm, 54.27 ± 6.64 kg)	Elite
Baena-Raya et al. [54]	12 (19.83 ± 4.2 years, 160.75 ± 8.37 cm, 57.64 ± 8.3 kg)	Sub-elite

Abbreviatures: NR (not reported).

**Table 3:** Methodological quality assessment.

Study	Question Number												Total Score
	1	2	3	4	6	7	10	11	12	16	18	20	
Benvenuti et al. [5]	1	1	1	1	1	1	1	1	1	1	1	1	12, high
Ramos-Campo et al. [7]	1	1	1	1	1	1	1	0	0	0	1	1	9, moderate
Barbero-Alvarez et al. [34]	1	1	1	1	1	1	1	0	0	0	1	1	9, moderate
Valladares-Rodríguez et al. [35]	1	1	1	0	1	1	1	1	1	0	1	1	10, high
Ünveren [36]	1	1	1	0	1	1	0	0	0	0	1	1	7, moderate
Teixeira et al. [37]	1	1	1	1	1	1	1	0	0	0	1	1	9, moderate
Teixeira et al. [38]	1	1	1	1	1	1	1	0	0	0	1	1	9, moderate
Supriadi et al. [39]	1	1	1	0	1	0	0	0	0	0	0	0	4, low
Souglis et al. [40]	1	1	1	1	1	1	1	0	0	0	1	1	9, moderate
Söğüt et al. [41]	1	1	1	0	1	1	1	0	0	0	1	1	8, moderate
Santos et al. [42]	1	1	1	1	1	1	1	1	1	0	1	1	11, high
Ruiz-Pérez et al. [43]	1	1	1	0	1	1	1	1	1	0	1	1	10, high
Purnamasari et al. [44]	1	1	1	1	1	1	1	0	0	0	1	1	9, moderate
Patti et al. [45]	1	1	1	1	1	1	1	1	1	1	1	1	12, high
Neves da Silva et al. [46]	1	1	1	1	1	1	0	0	0	0	1	1	8, moderate
Doewes et al. [47]	1	1	1	0	1	1	1	0	0	1	1	1	9, moderate
Lucas et al. [48]	1	1	1	1	1	1	1	1	1	0	1	1	11, high
Castillo et al. [49]	1	1	1	0	1	1	1	1	1	0	1	1	10, high
Canduela Valle et al. [50]	1	1	1	1	1	1	1	0	0	0	1	1	9, moderate
Ahmadi et al. [51]	1	1	1	1	1	1	1	0	0	0	1	1	9, moderate
Albalad-Aiguabella et al. [52]	1	1	1	0	1	1	1	0	0	0	1	1	8, moderate
Atakan et al. [53]	1	1	1	1	1	1	0	1	1	0	1	1	10, high
Baena-Raya et al. [54]	1	1	0	0	1	1	0	0	0	0	1	1	6, low

1 = yes, 0 = no or unable to determine (where applicable).

**Table 4:** Muscle strength.

Study	Competitive Level	Test/Protocol	Outcome (Unit)
Ramos-Campo et al. [7]	Elite and sub-elite	Isokinetic 60°/s	PTE DL Elite: 133.7 ± 23.4 (Nm) PTE DL Sub-Elite: 136.3 ± 25.5 (Nm) PTE NDL Elite: 137.3 ± 24.1 (Nm) PTE NDL Sub-elite: 143.3 ± 17.3 (Nm) PTF DL Elite: 77.0 ± 23.4 (Nm) PTF DL Sub-elite: 74.4 ± 11.6 (Nm) PTF NDL Elite: 70.2 ± 19.6 (Nm) PTF NDL Sub-elite: 71.3 ± 23.4 (Nm) H/Q ratio DL Elite: 0.6 ± 0.1 H/Q ratio DL Sub-elite: 0.5 ± 0.2 H/Q ratio NDL Elite: 0.6 ± 0.1 H/Q ratio NDL Sub-elite: 0.5 ± 0.2
Teixeira et al. [37]	Elite	Isokinetic 60°/s	KEcon: 148.8 ± 39.7 (Nm) KEcon: 131.7 ± 22.4 (Nm) KEecc: 263.6 ± 30.4 (Nm) KEecc: 247.7 ± 34.0 (Nm)
Purnamasari et al. [44]	Unspecified	1RM (squat test)	68.6 ± 9.21 (kg)
Ahmadi et al. [51]	Elite	Isokinetic 60°/s	RPTflx: 86.88 ± 11.73 (%) RPText: 140.54 ± 25.44 (%)
		Isokinetic 180°/s	RPTflx: 53.08 ± 19.80 (%) RPText: 87.55 ± 19.56 (%)
Atakan et al. [53]	Elite	Isometric (back and leg strength test)	80.0 ± 16.48 (kg)

Values expressed as mean ± SD; Abbreviations: KEcon (knee extensors concentric peak torque), KEecc (knee extensors eccentric peak torque), PTF (peak torque flexors), PTE (peak torque extensors), DL (dominant leg), NDL (non-dominant leg), H/Q ratio (hamstrings to quadriceps strength ratio) and RPT (relative peak torque).

**Table 5:** Jumping ability.

Study	Competitive Level	Test/Protocol	Outcome (Unit)
Ramos-Campo et al. [7]	Elite and sub-elite	CMJ	Elite: 26.7 ± 0.3 (cm)
			Sub-elite: 24.3 ± 0.3 (cm)
		SJ	Elite: 26.1 ± 0.4 (cm)
			Sub-elite: 24.2 ± 0.3 (cm)
Teixeira et al. [37]	Elite	SJ	29.6 ± 2.3 (cm)
		CMJ	33.1 ± 2.0 (cm)
Supriadi et al. [39]	Unspecified	VJ	35 (cm)
Souglis et al. [40]	Elite	CMJ	35.6 ± 2.9 (cm)
Söğüt et al. [41]	Unspecified	CMJ	25.5 ± 3.4 (cm)
Santos et al. [42]	Unspecified	CMJ	18.5 ± 1.7 (cm)
Ruiz-Pérez et al. [43]	Elite	CMJ	28 ± 0.3 (cm)
		DVJ	28 ± 0.5 (cm)
Patti et al. [45]	Elite	SJ	23.40 ± 1.68 (cm)
Neves da Silva et al. [46]	University	HJ	133.1 ± 22.4 (cm)
Doewes et al. [47]	Unspecified	VJ	50.45 ± 11.97 (cm)
Lucas et al. [48]	University	CMJ	25.69 ± 4.58 (cm)
Castillo et al. [49]	Elite	CMJ	31.47 ± 2.82 (cm)
		SJ	28.95 ± 2.57 (cm)
		ABK	33.60 ± 3.75 (cm)
Canduela Valle et al. [50]	Sub-elite	CMJ	23.9 ± 4.17 (cm)
Ahmadi et al. [51]	Elite	VJH	33.36 ± 0.99 (cm)
Albalad-Aiguabella et al. [52]	Sub-elite	CMJ	25.70 ± 4.44 (cm)
		SJ	20.83 ± 4.55 (cm)
		DVJ	24.71 ± 4.39 (cm)
		BJ	173 ± 18 (cm)

Values expressed as mean ± SD; Abbreviations: SJ (squat jump), CMJ (countermovement jump), VJ (vertical jump), DVJ (drop vertical jump), HJ (horizontal jump), ABK (Abalakov jump), VJH (Sargent’s jump height) and BJ (broad jump).

**Table 6:** Sprint performance.

Study	Competitive Level	Test/Protocol	Outcome (Unit)
Benvenuti et al. [5]	Regional	FIET	15.2 ± 0.5 (km/h)
Ramos-Campo et al. [7]	Elite and sub-elite	30 m	Elite: 4.9 ± 0.2 (s)
			Sub-elite: 5.0 ± 0.2 (s)
Barbero-Alvarez et al. [34]	Elite	20 m	3.89 ± 0.21 (s)
Valladares-Rodríguez et al. [35]	Elite	30–15 IFT	17.4 ± 1.3 (km/h)
Ünveren [36]	University	10 m	1.60 ± 0.11 (m/s)
		20 m	2.98 ± 0.20 (m/s)
		30 m	4.16 ± 0.39 (m/s)
Teixeira et al. [37]	Elite	40 m	40 m: 4.52 ± 0.13 (m/s)
Teixeira et al. [38]	Elite	FIET	14.69 ± 0.84 (km/h)
		RSA	9.39 ± 0.26 (s)
Supriadi et al. [39]	Unspecified	30 m	5.77 (s)
Souglis et al. [40]	Elite	10 m	1.97 ± 0.03 (s)
		20 m	3.49 ± 0.03 (s)
Ruiz-Pérez et al. [43]	Elite	5 m	1.15 ± 0.15 (s)
		10 m	1.99 ± 0.14 (s)
		15 m	2.71 ± 0.17 (s)
Purnamasari et al. [44]	Unspecified	20 m	5.339 ± 0.675 (s)
Doewes et al. [47]	Unspecified	20 m	3.76 ± 0.21 (s)
		30 m	5.63 ± 0.17 (s)
Lucas et al. [48]	University	20 m	20 m: 3.28 ± 0.27 (s)
Castillo et al. [49]	Elite	5 m	1.16 ± 0.08 (s)
		25 m	4.07 ± 0.15 (s)
		Yo-Yo	1120.00 ± 336.4 (m)
		RSA	3.87 ± 0.16 (s)
Atakan et al. [53]	Elite	5 m	1.14 ± 0.06 (s)
		10 m	1.98 ± 0.10 (s)
		15 m	2.72 ± 0.14 (s)
		25 m	4.26 ± 0.26 (s)
		RSA	4.26 ± 0.26 (s)
Baena-Raya et al. [54]	Sub-elite	10 m	2.06 ± 0.98 (s)
		20 m	3.43 ± 0.66 (s)
		30 m	4.99 ± 0.12 (s)

Values expressed as mean ± SD; Abbreviations: 30–15 IFT (30–15 intermittent fitness test), 5, 10, 15, 20, 25 and 30 m (meter acceleration of speed testing), FIET (futsal intermittent endurance test), RSA (repeated sprint ability) and Yo-Yo (Yo-Yo test).

**Table 7:** Change of direction ability and agility.

Study	Competitive Level	Test/Protocol	Outcome (Unit)
Benvenuti et al. [5]	Regional	RVS-T	17.32 ± 0.51 (s)
		PVS-T	14.69 ± 0.67 (s)
		DMT	2.63 ± 0.67 (s)
Ramos-Campo et al. [7]	Elite and sub-elite	AT	Elite: 5.5 (s)
			Sub-elite: 5.7 (s)
Ünveren [36]	University	IAT	16.99 ± 0.55 (s)
Supriadi et al. [39]	Unspecified	AT	13.72 (s)
Söğüt et al. [41]	Unspecified	505T	3.7 ± 0.3 (s)
Santos et al. [42]	Unspecified	IAT	19.6 ± 0.7 (s)
Ruiz-Pérez et al. [43]	Elite	IAT	16.97 ± 0.60 (s)
Patti et al. [45]	Elite	ATT	11.59 ± 1.34 (s)
Doewes et al. [47]	Unspecified	IAT	19.90 ± 0.71 (s)
Castillo et al. [49]	Elite	ATT	11.01 ± 0.31 (s)
Albalad-Aiguabella et al. [52]	Sub-elite	505MT R	2.84 ± 0.12 (s)
		505MT L	2.86 ± 0.15 (s)
		L-Run R	5.88 ± 0.31 (s)
		L-Run L	5.91 ± 0.32 (s)
		V-CUT	7.64 ± 0.50 (s)
Atakan et al. [53]	Elite	IAT	16.77 ± 0.34 (s)
Baena-Raya et al. [54]	Sub-elite	505MT	2.82 ± 0.19 (s)
		505	4.45 ± 0.31 (s)
		V-CUT	7.25 ± 0.58 (s)

Values expressed as mean ± SD; Abbreviations: IAT (Illinois agility test), RSA (repeated sprint ability), L-Run (L-run test), R (right), L (left), 505 (505 test), V-CUT (V-cut test), AT (agility test), ATT (agility *t*-test), 505MT (505 modified test), RVS-T (reactive visual stimuli agility test), PVS-T (planned visual stimuli agility test) and DMT (decision-making time).

## Data Availability

All data analyzed in this study were obtained from previously published studies, as cited in the manuscript.

## Supplementary Materials

The following supporting information can be downloaded at: https://www.mdpi.com/article/10.3390/sports14030098/s1, File S1: Search strategy; File S2: PRISMA 2020 abstract checklist; File S3: PRISMA 2020 checklist.

## Abbreviations

The following abbreviations are used in this manuscript:

FIFA	Fédération Internationale de Football Association
CoD	Change of Direction
PRISMA	Preferred Reporting Items for Systematic Reviews and Meta-Analyses
PROSPERO	International Prospective Register of Systematic Reviews
MeSH	Medical Subject Heading
1RM	One Repetition Maximum
CMJ	Countermovement Jump
SJ	Squat Jump
DJ	Drop Jump
BJ	Broad Jump
ABK	Abalakov Jump
VJH	Sargent’s Jump Height
VJ	Vertical Jump
HJ	Horizontal Jump
DVJ	Drop Vertical Jump
IAT	Illinois Agility Test

## References

[1] Naser N., Ali A., Macadam P. (2017). Physical and physiological demands of futsal. J. Exerc. Sci. Fit..

[2] Matzenbacher F., Pasquarelli B.N., Rabelo F.N., Stanganelli L.C.R. (2014). Demanda fisiológica no futsal competitivo. Características físicas e fisiológicas de atletas profissionais. Rev. Andal. Med. Deporte.

[3] Barbero-Alvarez J.C., Soto V.M., Barbero-Alvarez V., Granda-Vera J. (2008). Match analysis and heart rate of futsal players during competition. J. Sports Sci..

[4] Beato M., Coratella G., Schena F. (2016). Brief review of the state of art in futsal. J. Sports Med. Phys. Fit..

[5] Benvenuti C., Minganti C., Condello G., Capranica L., Tessitore A. (2010). Agility assessment in female futsal and soccer players. Medicina.

[6] Barreira J., Wunderlich K., Batista A.B.C.V., Silva Junior J.E.P.d. (2025). Physiological demands and player characteristics in women’s futsal: A systematic review. Front. Physiol..

[7] Ramos-Campo D.J., Rubio-Arias J.A., Carrasco-Poyatos M., Alcaraz P.E. (2016). Physical performance of elite and subelite Spanish female futsal players. Biol. Sport.

[8] Rebelo M., Marques C., Crisóstomo R., Batista M., Paulo R., Rocha J., Serrano J. (2024). The Influence of Futsal Players’ Initial Physical Condition on the Occurrence of Injuries. Int. J. Sports Med..

[9] Sekulic D., Foretic N., Gilic B., Esco M.R., Hammami R., Uljevic O., Versic S., Spasic M. (2019). Importance of Agility Performance in Professional Futsal Players; Reliability and Applicability of Newly Developed Testing Protocols. Int. J. Environ. Res. Public Health.

[10] Gepfert M., Gołaś A., Drozd M., Strońska-Grabień K., Sawicki P., Lulińska E. (2025). Physical performance differences between top and first league female football players: In-sights from locomotion metrics. Balt. J. Health Phys. Act..

[11] Liu C., Peng W., Qu W., Zhang Z., Sun J., He J., Cheng B., Li D. (2025). Gender differences in the impact of fatigue on lower limb landing biomechanics and their association with anterior cruciate ligament (ACL) injuries: A systematic review and meta-analysis. PLoS ONE.

[12] Sell T.C., Lephart S.M., Noyes F.R., Barber-Westin S. (2018). Neuromuscular Differences Between Men and Women. ACL Injuries in the Female Athlete: Causes, Impacts, and Conditioning Programs.

[13] Amdi C.H., Fyfe J., Yoon S., Nuckols G., Refalo M. (2025). Biological sex differences in fatigue in resistance-trained individuals: A scoping review. Int. J. Sports Med..

[14] Fort-Vanmeerhaeghe A., Pujol-Marzo M., Milà R., Campos B., Nevot-Casas O., Casadevall-Sayeras P., Peña J. (2025). Injury Risk and Overall Well-Being During the Menstrual Cycle in Elite Adolescent Team Sports Athletes. Healthcare.

[15] Niering M., Wolf-Belala N., Seifert J., Tovar O., Coldewey J., Kuranda J., Muehlbauer T. (2024). The Influence of Menstrual Cycle Phases on Maximal Strength Performance in Healthy Female Adults: A Systematic Review with Meta-Analysis. Sports.

[16] Mattu A.T., Ghali B., Linton V., Zheng A., Pike I. (2022). Prevention of Non-Contact Anterior Cruciate Ligament Injuries among Youth Female Athletes: An Umbrella Review. Int. J. Environ. Res. Public Health.

[17] Fernández-Galván L.M., Hernández Santana C., López-Nuevo C., Sánchez-Infante J. (2024). Injuries in Female Futsal Players: A Systematic Review. Sports.

[18] Barreira J., Silva Junior J.E.P.d., de Souza C.P. (2024). Research on women’s futsal: A scoping review. Sci. Med. Footb..

[19] Sanmiguel-Rodríguez A. (2021). Presence of women in futsal. A systematic review. Arch. Med. Deporte.

[20] Albalad-Aiguabella R., Vicente-Rodríguez G., Muniz-Pardos B., Roso-Moliner A., Villanueva-Guerrero O., Mainer-Pardos E. (2025). Associations Between Physical Performance and Asymmetry in Jump, Change of Direction, and Dorsiflexion Tests in Adult Elite Female Futsal Players. Appl. Sci..

[21] Galy O., Zongo P., Chamari K., Chaouachi A., Michalak E., Dellal A., Castagna C., Hue O. (2015). Anthropometric and physiological characteristics of Melanesian futsal players: A first approach to talent identification in Oceania. Biol. Sport.

[22] Ayarra R., Nakamura F.Y., Iturricastillo A., Castillo D., Yanci J. (2018). Differences in Physical Performance According to the Competitive Level in Futsal Players. J. Hum. Kinet..

[23] Nakamura F.Y., Pereira L.A., Cal Abad C.C., Kobal R., Kitamura K., Roschel H., Rabelo F., Souza W.A., Loturco I. (2016). Differences in physical performance between U-20 and senior top-level Brazilian futsal players. J. Sports Med. Phys. Fit..

[24] Rebelo M., Marques C., Crisóstomo R., Batista M., Paulo R., Duarte-Mendes P., Honório S., Serrano J. (2025). Índices de composición corporal, fuerza y potencia muscular en los diferentes niveles competitivos del fútbol sala. Retos.

[25] Page M.J., McKenzie J.E., Bossuyt P.M., Boutron I., Hoffmann T.C., Mulrow C.D., Shamseer L., Tetzlaff J.M., Akl E.A., Brennan S.E. (2021). The PRISMA 2020 statement: An updated guideline for reporting systematic reviews. BMJ.

[26] Khan K.S. (2003). Systematic Reviews to Support Evidence-Based Medicine: How to Review and Apply Findings of Healthcare Research.

[27] Ouzzani M., Hammady H., Fedorowicz Z., Elmagarmid A. (2016). Rayyan—A web and mobile app for systematic reviews. Syst. Rev..

[28] Harrison H., Griffin S.J., Kuhn I., Usher-Smith J.A. (2020). Software tools to support title and abstract screening for systematic reviews in healthcare: An evaluation. BMC Med. Res. Methodol..

[29] Spyrou K., Freitas T.T., Marín-Cascales E., Alcaraz P.E. (2020). Physical and Physiological Match-Play Demands and Player Characteristics in Futsal: A Systematic Review. Front. Psychol..

[30] Cummins C., Orr R., O’Connor H., West C. (2013). Global positioning systems (GPS) and microtechnology sensors in team sports: A systematic review. Sports Med..

[31] Elliott-Sale K.J., McNulty K.L., Ansdell P., Goodall S., Hicks K.M., Thomas K., Swinton P.A., Dolan E. (2020). The Effects of Oral Contraceptives on Exercise Performance in Women: A Systematic Review and Meta-analysis. Sports Med..

[32] Mikkonen R.S., Ihalainen J.K., Hackney A.C., Häkkinen K. (2024). Perspectives on Concurrent Strength and Endurance Training in Healthy Adult Females: A Systematic Review. Sports Med..

[33] Whitehead S., Till K., Weaving D., Jones B. (2018). The Use of Microtechnology to Quantify the Peak Match Demands of the Football Codes: A Systematic Review. Sports Med..

[34] Barbero-Alvarez J.C., Subiela J.V., Granda-Vera J., Castagna C., Gómez M., Del Coso J. (2015). Aerobic fitness and performance in elite female futsal players. Biol. Sport.

[35] Valladares-Rodríguez S., Rey E., Mecías-Calvo M., Barcala-Furelos R., Bores-Cerezal A.J. (2017). Reliability and Usefulness of the 30-15 Intermittent Fitness Test in Male and Female Professional Futsal Players. J. Hum. Kinet..

[36] Ünveren A. (2015). Investigating Women Futsal and Soccer Players’ Acceleration, Speed and Agility Features. Anthropologist.

[37] Teixeira A., Arins F., De Lucas R., Nakamura F.Y., Loturco I., Guglielmo L. (2018). Shuttle-Run Interval Training with More Directional Changes Induces Superior Gains in Shuttle Sprint Performance in Female Professional Futsal Players. Hum. Mov..

[38] Teixeira A.S., Arins F.B., De Lucas R.D., Carminatti L.J., Dittrich N., Nakamura F.Y., Guglielmo L.G.A. (2019). Comparative Effects of Two Interval Shuttle-Run Training Modes on Physiological and Performance Adaptations in Female Professional Futsal Players. J. Strength Cond. Res..

[39] Supriadi D., Friskawati G., Karisman V. (2023). Physical Fitness of Futsal Athletes in Competition Preparation. Int. J. Hum. Mov. Sports Sci..

[40] Souglis A., Bourdas D.I., Gioldasis A., Ispirlidis I., Philippou A., Zacharakis E., Apostolidis A., Efthymiou G., Travlos A.K. (2023). Time Course of Performance Indexes, Oxidative Stress, Inflammation, and Muscle Damage Markers after a Female Futsal Match. Sports.

[41] Söğüt M., Yapici H., Luz L.G., Giudicelli B., Clemente F.M., Doğan A.A. (2022). Maturity-associated variations in anthropometry, physical fitness, and sport-specific skills among young male and female futsal players. Hum. Mov..

[42] Santos I.A.D., Lemos M.P., Coelho V.H.M., Zagatto A.M., Marocolo M., Soares R.N., Neto O.B., Mota G.R. (2020). Acute Photobiomodulation Does Not Influence Specific High-Intensity and Intermittent Performance in Female Futsal Players. Int. J. Environ. Res. Public Health.

[43] Ruiz-Pérez I., Raya-González J., López-Valenciano A., Robles-Palazón F.J., Ayala F. (2023). Physical Differences between Injured and Non-Injured Elite Male and Female Futsal Players. Appl. Sci..

[44] Purnamasari R., Purnamasari I., Novian G. (2025). The Effect of Contrast Training Methods on the Speed and Lower Body Strength of Futsal Athletes. Int. J. Hum. Mov. Sports Sci..

[45] Patti A., Giustino V., Cataldi S., Stoppa V., Ferrando F., Marvulli R., Farì G., Neşe Ş.F., Bianco A., Muscella A. (2022). Effects of 5-Week of FIFA 11+ Warm-Up Program on Explosive Strength, Speed, and Perception of Physical Exertion in Elite Female Futsal Athletes. Sports.

[46] Neves da Silva V.F., Aguiar S.D.S., Sousa C.V., Sotero R.D.C., Filho J.M.S., Oliveira I., Mota M.R., Simões H.G., Sales M.M. (2017). Effects of short-term plyometric training on physical fitness parameters in female futsal athletes. J. Phys. Ther. Sci..

[47] Doewes R., Elumalai G., Azmi S. (2022). Differences In Speed, Acceleration, Agility, and Power of The Leg Muscles In Female Futsal And Football Players. Rev. Iberoam. Psicol. Ejerc. Y El Deporte.

[48] Lucas G., Sá S., Pinheiro B., Godinho I., Casanova F., Reis V., Garrido N., Vilaça-Alves J. (2024). Comparison between Warm-Up Protocols in Post-Activation Potentiation Enhancement (PAPE) ofSprint and Vertical Jump Performance in a FemaleFutsal Team. Res. Q. Exerc. Sport.

[49] Castillo M., Martínez-Sanz J.M., Penichet-Tomás A., Sellés S., González-Rodriguez E., Hurtado-Sánchez J.A., Sospedra I. (2022). Relationship between Body Composition and Performance Profile Characteristics in Female Futsal Players. Appl. Sci..

[50] Canduela Valle S., Osmani F., Lago Fuentes C. (2023). Propuesta preventiva sobre el esguince de tobillo en jugadoras de 2ªRFEF Futsal. RICYDE Rev. Int. Cienc. Deporte.

[51] Ahmadi M., Hoorang N., Imanian B., Hemmatinafar M., Rezaei R., Nemati J., Eftekhari F., Alkasasbeh W.J. (2024). Boosting Recovery: Omega-3 and Whey Protein Enhance Strength and Ease Muscle Soreness in Female Futsal Players. Nutrients.

[52] Albalad-Aiguabella R., Mainer-Pardos E., Roso-Moliner A., Lozano D., Vicente-Rodríguez G., Muniz-Pardos B. (2025). Fitness Profiles of Highly Trained Female Futsal Players According to Their Playing Positions. Int. J. Sports Physiol. Perform..

[53] Atakan M.M., Karavelioğlu M.B., Harmancı H., Cook M., Bulut S. (2019). Short term creatine loading without weight gain improves sprint, agility and leg strength performance in female futsal players. Sci. Sports.

[54] Baena-Raya A., García-Ortega D., Sánchez-López S., Soriano-Maldonado A., Reyes P.J., Rodríguez-Pérez M.A. (2021). The Influence of Sprint Mechanical Properties on Change of Direction in Female Futsal Players. J. Hum. Kinet..

[55] Brígido-Fernández I., García-Muro San José F., Charneco-Salguero G., Cárdenas-Rebollo J.M., Ortega-Latorre Y., Carrión-Otero O., Fernández-Rosa L. (2022). Knee Isokinetic Profiles and Reference Values of Professional Female Soccer Players. Sports.

[56] Quigley T., Greig M. (2025). The influence of menstrual cycle phase on isokinetic knee flexor and extensor strength in female soccer players: A pilot study. Res. Sports Med..

[57] Zhang Q., Léam A., Fouré A., Wong D.P., Hautier C.A. (2021). Relationship Between Explosive Strength Capacity of the Knee Muscles and Deceleration Performance in Female Professional Soccer Players. Front. Physiol..

[58] Zabaloy S., Villaseca-Vicuña R., Giráldez J., Alcaraz P.E., Filter-Ruger A., Freitas T.T., Loturco I. (2022). Body composition and physical performance measures in elite female football players: Differences across playing positions and associations with kicking velocity and curve sprint performance. Mov. Sport Sci. Mot..

[59] Vescovi J.D., Rupf R., Brown T.D., Marques M.C. (2011). Physical performance characteristics of high-level female soccer players 12–21 years of age. Scand. J. Med. Sci. Sports.

[60] França C., Saldanha C., Martins F., de Maio Nascimento M., Marques A., Ihle A., Sarmento H., Campos P., Gouveia É.R. (2025). Lower body strength and body composition in female football. Sci. Rep..

[61] Sedano S., Vaeyens R., Philippaerts R.M., Redondo J.C., Cuadrado G. (2009). Anthropometric and anaerobic fitness profile of elite and non-elite female soccer players. J. Sports Med. Phys. Fit..

[62] Andersen E., Lockie R.G., Dawes J.J. (2018). Relationship of Absolute and Relative Lower-Body Strength to Predictors of Athletic Performance in Collegiate Women Soccer Players. Sports.

[63] Haugen T.A., Tønnessen E., Seiler S. (2012). Speed and countermovement-jump characteristics of elite female soccer players, 1995–2010. Int. J. Sports Physiol. Perform.

[64] Chattanta V., Verma N., Mehra P. (2024). Quantifying the difference between male and female agility in football players: A cross-sectional study. Phys. Ther. Sport.

