# Supplementary material for: Neuromuscular Characteristics of Female Futsal Players: A Systematic Review
Source: Sports (Basel). 2026 Mar 3;14(3):98. doi: 10.3390/sports14030098 (PMC13030330; doi:10.3390/sports14030098)
Supplement: Supplementary file 1 [file sports-14-00098-s001.zip › Supplementary File S3-PRISMA_2020_checklist.pdf]

PRISMA 2020 Checklist

Section and Topic	Item #	Checklist item	Location where item is reported
**TITLE**			
Title	1	Identify the report as a systematic review.	Yes. Title
**ABSTRACT**			
Abstract	2	See the PRISMA 2020 for Abstracts checklist.	Yes.
**INTRODUCTION**			
Rationale	3	Describe the rationale for the review in the context of existing knowledge.	Introduction, L63-67
Objectives	4	Provide an explicit statement of the objective(s) or question(s) the review addresses.	Introduction, L78-82
**METHODS**			
Eligibility criteria	5	Specify the inclusion and exclusion criteria for the review and how studies were grouped for the syntheses.	Clearly described in section 2.3, based on the PECOS strategy. Well-defined exclusion criteria (gender, study type, etc.). Grouping by competitive level and neuromuscular variables.
Information sources	6	Specify all databases, registers, websites, organisations, reference lists and other sources searched or consulted to identify studies. Specify the date when each source was last searched or consulted.	Databases: PubMed, Scopus, and SPORTDiscus, searched between April and May 2025, with no year restrictions.
Search strategy	7	Present the full search strategies for all databases, registers and websites, including any filters and limits used.	Fully described in section 2.2 with a combination of MeSH terms and Boolean operators.
Selection process	8	Specify the methods used to decide whether a study met the inclusion criteria of the review, including how many reviewers screened each record and each report retrieved, whether they worked independently, and if applicable, details of automation tools used in the process.	Section 2.4: Two reviewers (CM and MR) independently analysed titles and abstracts using Rayyan, which also removes duplicates and facilitates consensus.
Data collection process	9	Specify the methods used to collect data from reports, including how many reviewers collected data from each report, whether they worked independently, any processes for obtaining or confirming data from study investigators, and if applicable, details of automation tools used in the process.	Section 2.5: Two reviewers (CM and MR) extracted the data independently using a standardised table in Excel. Variables clearly described.
Data items	10a	List and define all outcomes for which data were sought. Specify whether all results that were compatible with each outcome domain in each study were sought (e.g. for all measures, time points, analyses), and if not, the methods used to decide which results to collect.	Section 2.5: Data were collected on muscle strength, jumping, speed, agility, and change of direction.
	10b	List and define all other variables for which data were sought (e.g. participant and intervention characteristics, funding sources). Describe any assumptions made about any missing or unclear information.	Also in 2.5: age, height, body mass, competitive level. Absence of data not mentioned.
Study risk of bias assessment	11	Specify the methods used to assess risk of bias in the included studies, including details of the tool(s) used, how many reviewers assessed each study and whether they worked independently, and if applicable, details of automation tools used in the process.	Section 2.6: Independent assessment by two reviewers using a modified Downs & Black scale (12 items, 3 domains).

PRISMA 2020 Checklist

Section and Topic	Item #	Checklist item	Location where item is reported
Effect measures	12	Specify for each outcome the effect measure(s) (e.g. risk ratio, mean difference) used in the synthesis or presentation of results.	Mean ± standard deviation (SD) for each variable.
Synthesis methods	13a	Describe the processes used to decide which studies were eligible for each synthesis (e.g. tabulating the study intervention characteristics and comparing against the planned groups for each synthesis (item #5)).	Included studies were grouped by neuromuscular variables (strength, jump, speed, agility, and change of direction) and, when possible, by competitive level, following the predefined PECOS criteria.
	13b	Describe any methods required to prepare the data for presentation or synthesis, such as handling of missing summary statistics, or data conversions.	Section 2.7: All results are presented as mean ± standard deviation (SD).
	13c	Describe any methods used to tabulate or visually display results of individual studies and syntheses.	Results section, table 2.
	13d	Describe any methods used to synthesize results and provide a rationale for the choice(s). If meta-analysis was performed, describe the model(s), method(s) to identify the presence and extent of statistical heterogeneity, and software package(s) used.	Section 2.7: No meta-analysis was performed due to heterogeneity; a descriptive and narrative synthesis was chosen instead. Data grouped by variable and competitive level.
	13e	Describe any methods used to explore possible causes of heterogeneity among study results (e.g. subgroup analysis, meta-regression).	No formal heterogeneity analyses were conducted, as no meta-analysis was performed. Differences between studies were described narratively according to variables and competitive levels.
	13f	Describe any sensitivity analyses conducted to assess robustness of the synthesized results.	Not mentioned.
Reporting bias assessment	14	Describe any methods used to assess risk of bias due to missing results in a synthesis (arising from reporting biases).	Not mentioned.
Certainty assessment	15	Describe any methods used to assess certainty (or confidence) in the body of evidence for an outcome.	Not mentioned.
**RESULTS**			
Study selection	16a	Describe the results of the search and selection process, from the number of records identified in the search to the number of studies included in the review, ideally using a flow diagram.	The process is illustrated in the PRISMA flowchart (Figure 1).
	16b	Cite studies that might appear to meet the inclusion criteria, but which were excluded, and explain why they were excluded.	Twenty-four studies were excluded after full text review because they did not meet the inclusion criteria (e.g., gender not specified, data combined with men, mixed results with other modalities).
Study characteristics	17	Cite each included study and present its characteristics.	Table 2.

Section and Topic	Item #	Checklist item	Location where item is reported
Risk of bias in studies	18	Present assessments of risk of bias for each included study.	Methodological quality was assessed using the modified Downs & Black scale. Scores ranged from 4 to 12 (low to high), as shown in Table 3.
Results of individual studies	19	For all outcomes, present, for each study: (a) summary statistics for each group (where appropriate) and (b) an effect estimate and its precision (e.g. confidence/credible interval), ideally using structured tables or plots.	Individual results are presented in Tables 2 and 3, including means ± standard deviation for strength, jump, speed, agility, and CoD, stratified by competitive level.
Results of syntheses	20a	For each synthesis, briefly summarise the characteristics and risk of bias among contributing studies.	Narrative syntheses were performed by neuromuscular variable (strength, jump, sprint, agility/CoD). Most studies had moderate to high methodological quality.
	20b	Present results of all statistical syntheses conducted. If meta-analysis was done, present for each the summary estimate and its precision (e.g. confidence/credible interval) and measures of statistical heterogeneity. If comparing groups, describe the direction of the effect.	No meta-analysis was performed due to methodological heterogeneity between studies.
	20c	Present results of all investigations of possible causes of heterogeneity among study results.	No formal analyses of heterogeneity were performed; differences between studies were described narratively.
	20d	Present results of all sensitivity analyses conducted to assess the robustness of the synthesized results.	No sensitivity analyses were performed, given the descriptive nature of the review.
Reporting biases	21	Present assessments of risk of bias due to missing results (arising from reporting biases) for each synthesis assessed.	No formal assessment of publication bias was performed, as there was no meta-analysis.
Certainty of evidence	22	Present assessments of certainty (or confidence) in the body of evidence for each outcome assessed.	The certainty of the evidence was not formally assessed due to the observational and heterogeneous nature of the included studies.
**DISCUSSION**			
Discussion	23a	Provide a general interpretation of the results in the context of other evidence.	Yes. The results indicate that the neuromuscular profile of futsal players is similar to that of athletes in other intermittent team sports, especially women's football. The ability to change direction stands out as a possible specific and discriminating characteristic in women's futsal.

PRISMA

PRISMA 2020 Checklist

Section and Topic	Item #	Checklist item	Location where item is reported
	23b	Discuss any limitations of the evidence included in the review.	Yes. The main limitation of the available evidence is the small number of studies on women's futsal, often with small samples and heterogeneous protocols, which makes direct comparisons and generalisations difficult.
	23c	Discuss any limitations of the review processes used.	The review was restricted to three databases (PubMed, Scopus, and SPORTDiscus) and studies published in three languages, and there may have been omissions of grey literature or unpublished data.
	23d	Discuss implications of the results for practice, policy, and future research.	Conclusion section. The results may support coaches and physical trainers in prescribing more specific training programmes for women's futsal. Future research should standardise protocols, include larger samples, and explore the relationship between neuromuscular variables and competitive performance.
**OTHER INFORMATION**			
Registration and protocol	24a	Provide registration information for the review, including register name and registration number, or state that the review was not registered.	PROSPERO CRD420251055503
	24b	Indicate where the review protocol can be accessed, or state that a protocol was not prepared.	The review protocol is available at PROSPERO under the registration ID.
	24c	Describe and explain any amendments to information provided at registration or in the protocol.	No amendments to the registered protocol were made
Support	25	Describe sources of financial or non-financial support for the review, and the role of the funders or sponsors in the review.	This research was supported by the Foundation for Science and Technology, I.P. (Portugal), within the scope of SPRINT - Sport Physical Activity and Health Research & Innovation Center (UID/6185/2023).
Competing interests	26	Declare any competing interests of review authors.	No potential conflict of interest was reported by the author(s)
Availability of data, code and	27	Report which of the following are publicly available and where they can be found: template data collection forms; data extracted from included studies; data used for all analyses; analytic code; any other materials used in the review.	This study did not generate any new data beyond that included in

PRISMA 2020 Checklist

Section and Topic	Item #	Checklist item	Location where item is reported
other materials			the articles analyzed.

From: Page MJ, McKenzie JE, Bossuyt PM, Boutron I, Hoffmann TC, Mulrow CD, et al. The PRISMA 2020 statement: an updated guideline for reporting systematic reviews. BMJ 2021;372:n71. doi: 10.1136/bmj.n71. This work is licensed under CC BY 4.0. To view a copy of this license, visit https://creativecommons.org/licenses/by/4.0/
