# Supplementary material for: Neuromuscular Characteristics of Female Futsal Players: A Systematic Review
Source: Sports (Basel). 2026 Mar 3;14(3):98. doi: 10.3390/sports14030098 (PMC13030330; doi:10.3390/sports14030098)
Supplement: Supplementary file 1 [file sports-14-00098-s001.zip › Supplementary File S1-Search strategy.pdf]

**Search strategy by databases**

PubMed:

("futsal" OR "indoor soccer") AND ("female" OR "women") AND ("physical performance" OR "physical fitness" OR "strength" OR "agility" OR "neuromuscular" OR "power" OR "speed" OR "endurance" OR "jump").

Scopus:

TITLE-ABS-KEY (futsal OR "indoor soccer") AND TITLE-ABS-KEY (female OR women) AND TITLE-ABS-KEY ("physical performance" OR "physical condition" OR strength OR agility OR neuromuscular OR speed OR power OR endurance OR jump).

SPORTDiscus:

(futsal OR "indoor soccer") AND (female OR women) AND ("physical performance" OR "physical fitness" OR strength OR agility OR neuromuscular OR speed OR power OR endurance OR jump).
